# Genetic and Environmental Factors Influencing the Placental Growth Factor (PGF) Variation in Two Populations

**DOI:** 10.1371/journal.pone.0042537

**Published:** 2012-08-20

**Authors:** Rossella Sorice, Daniela Ruggiero, Teresa Nutile, Mario Aversano, Lotte Husemoen, Allan Linneberg, Catherine Bourgain, Anne-Louise Leutenegger, Marina Ciullo

**Affiliations:** 1 Institute of Genetics and Biophysics A. Buzzati-Traverso, CNR, Naples, Italy; 2 Research Centre for Prevention and Health, Glostrup, Denmark; 3 Inserm/University Paris SUD U669, Villejuif, France; 4 INSERM, U946, Paris, France; Kunming Institute of Zoology, Chinese Academy of Sciences, China

## Abstract

Placental Growth Factor (PGF) is a key molecule in angiogenesis. Several studies have revealed an important role of PGF primarily in pathological conditions (e.g.: ischaemia, tumour formation, cardiovascular diseases and inflammatory processes) suggesting its use as a potential therapeutic agent. However, to date, no information is available regarding the genetics of PGF variability. Furthermore, even though the effect of environmental factors (e.g.: cigarette smoking) on angiogenesis has been explored, no data on the influence of these factors on PGF levels have been reported so far. Here we have first investigated PGF variability in two cohorts focusing on non-genetic risk factors: a study sample from two isolated villages in the Cilento region, South Italy (N = 871) and a replication sample from the general Danish population (N = 1,812). A significant difference in PGF mean levels was found between the two cohorts. However, in both samples, we observed a strong correlation of PGF levels with ageing and sex, men displaying PGF levels significantly higher than women. Interestingly, smoking was also found to influence the trait in the two populations, although differently. We have then focused on genetic risk factors. The association between five single nucleotide polymorphisms (SNPs) located in the *PGF* gene and the plasma levels of the protein was investigated. Two polymorphisms (rs11850328 and rs2268614) were associated with the PGF plasma levels in the Cilento sample and these associations were strongly replicated in the Danish sample. These results, for the first time, support the hypothesis of the presence of genetic and environmental factors influencing PGF plasma variability.

## Introduction

Placental growth factor (PGF), originally identified in the placenta, is a protein highly homologous to vascular endothelial growth factor (VEGF) [1]. As the specific ligand of the VEGF receptor 1 (Flt-1), PGF has potent angiogenic properties and also induces the growth and migration of the endothelial cells (EC) [2]. The mechanism of the PGF function during angiogenesis has been firmly established: PGF directly stimulates the ECs, affects the monocyte functions and synergistically amplifies the action of VEGF [3].

PGF plays important roles in the maternal vascular function and the remodelling of the maternal vasculature during pregnancy including the uterine spiral arteries which pervade the placenta. Decreased levels of PGF during midgestation have been shown to be predictive of the subsequent development of clinical pre-eclampsia [4]. Although PGF expression is normally low in most other organs/tissues, the expression is inducible and required in tissues undergoing pathological angiogenesis [5] including vascularized tumours [6], [7], [8].

PGF overexpression produces abundant angiogenesis in normal mouse tissues. Recent studies have demonstrated that mice deficient in PGF or with an inhibition of VEGFR-1 exhibited an impaired collateral artery growth in the limbs and the neovascularization of tumours, choroids and ischaemic retinas, and that exogenous PGF delivery promoted angiogenesis or collateral artery growth in ischaemic limbs, hearts and skin [9], [10], [11], [12], [13]. Clinical studies have shown that PGF levels positively correlate with a poor prognosis for various types of cancer, including hepatocellular, colorectal, renal and other cancers [14], [15]. Recently, several studies have suggested the role of PGF as a proatherogenic cytokine and new biomarker of cardiovascular events. The analysis carried out in the Nurses Health study described PGF as an independent predictor of coronary heart diseases after adjustment for several confounding factors (age, smoking, parental history of myocardial infarction, physical activity, alcohol intake and BMI), suggesting that PGF might be involved in the formation of atherosclerotic lesions [16]. Again, high PGF levels have been associated with adverse long-term outcomes in patients with acute coronary syndrome [17] and an elevated expression of PGF has been shown to contribute to inflammation in rheumatoid arthritis by triggering the production of pro-inflammatory cytokines [18].

All these data show that the amount of PGF has a relevant impact in the determination of pathological conditions. The PGF studies carried out so far have involved specific, clinically selected, samples. Little data exist on the PGF plasma variation in general populations and no data are available regarding the extent of genetic influences on PGF plasma variation. In this work, we have explored the influence of factors, both genetic and environmental, on the PGF levels in two population samples of European descent: the “Cilento sample” as the study sample, recruited in two isolated villages in South Italy, and the “Denmark sample” as the replication sample, recruited in the general Danish population.

## Materials and Methods

### Sample characteristics

#### Cilento population-based sample

The Cilento study includes 871 subjects recruited through a population-based sampling strategy in two small isolated villages of the Cilento region, South Italy: 556 individuals from the village of Gioi and 315 from the village of Cardile. Even though these two villages are located 7 km apart, the genealogy reconstruction showed that inhabitants from the two villages are related to each other and all included in a unique large pedigree of 5,272 members connecting the 871 individuals of the Cilento sample [19]. The individuals participating in the study were aged from 15 to 103 years.

The study design was approved by the ethics committee of Azienda Sanitaria Locale Napoli 1. The study was conducted according to the criteria established by the declaration of Helsinki and each subject signed an informed consent before participating in the study.

#### The MONICA 10 sample

The replication sample consists of 1,812 subjects from a general population of European origin (Denmark) and included in the MONICA 10 project. The recruitment and characteristics of this cohort have been described elsewhere [20]. Briefly, the MONICA 10 project is a prospective study established in the early 1980s with the aim of studying cardiovascular diseases and their risk factors over a ten year period. In the Denmark study sample the subjects are classified into 10-year age groups and cover a range of ages from 40 to 73 years. They were randomly chosen in a defined area of Copenhagen and invited to a health examination in 1982–4 and then re-examined in 1993–4.

### Phenotype characterization

In both samples the participants completed a self-administered questionnaire concerning their past and current medical history, previous surgery, current diseases and pharmacological treatments and lifestyle. With regard to smoking behaviour, only “smoker” and “never smoker” categories were included in the study. “Smokers” were defined as individuals who reported smoking on a daily or weekly basis. “Never smokers” included individuals who reported no regular smoking in the past until the date of the interview. Any former and occasional smokers were excluded from this study. Menstruation variable was defined on the basis of self-reported data. Post-menopausal women and men were included in the same category.

### Blood sampling and PGF measurements

Blood samples were collected in the morning after the participants had been fasting for at least 12 h. Aliquots of plasma were immediately prepared and stored at −80°C, and were subsequently used for the assessment of GF levels.

PGF (pg/ml) was measured using an electrochemiluminescence immunoassay on the Elecsys2010 analyzer (Roche Diagnostics, Mannheim, Germany). Pregnant women were excluded from the study because of their high level of PGF in the plasma. PGF was log-transformed before testing for any association.

The Denmark sample is based on measurements of PGF in serum samples obtained in the re-examination in 1993–4.

### Genotyping and quality control

Genomic DNA was extracted from 10 ml of peripheral blood by the Flexigene kit (Qiagen) following the manufacturer's instructions. Single nucleotide polymorphisms were genotyped using the TaqMan SNP genotyping assay and the SDS software v2.1 was used for allele discrimination (Applied Biosystems, Foster City, CA, USA). The Danish genotyping was performed by Kbioscience, Hoddesdon, UK applying the same methodology. The five SNPs in the PGF gene region were selected because they cover the two linkage disequilibrium blocks of the PGF gene region according to the CEU data from the HapMap database (the rs11850328 and rs2268614 variants are located in one haploblock and the rs12411, rs8185 and rs2268613 polymorphisms are placed in the other). Considering that these variants were the unique SNPs in this region for which a genotyping assay was available at the beginning of the study, we decided to genotype all the five SNPs. All genotypic distributions were in the Hardy-Weinberg (HW) equilibrium (p<0.05). The HW equilibrium was assessed using the Haploview v. 4.1 program (http://www.broad.mit.edu/mpg/haploview/). The rate of successful genotypes was above 90% for each SNP in both the Cilento and the Denmark samples. As the *PGF* gene is on the reverse strand, all the SNP genotypes described in this study are referred to this strand. In the Cilento data, Mendelian inheritance inconsistencies were checked with the Pedcheck program [21].

### Statistical analyses

Because PGF levels are modified in inflammatory and cardiovascular diseases, a binary variable, called the “*disease status*”, was created to take into account information about the following diseases: hypertension, diabetes, atherosclerosis (evaluated according to B-mode ultrasound measurements of intima-media thickness (IMT) of the common, internal and external carotid and carotid bifurcation. Atherosclerosis was defined for IMTmax (the maximum among the IMT measurements) values greater than 1.3 mm), cardiac hypertrophy (defined on the basis of the left ventricular mass (LVM) index greater than 110 g/m2 and 135 g/m2 for women and men respectively. LVM was determined by echocardiography according to the Penn Convention), obesity (defined both on BMI and on waist circumference measurements), metabolic syndrome, chronic kidney disease, cancer and cardiovascular diseases. The quantitative variables were dichotomized to generate the “*disease status*” variable. In this context individuals having at least one of the selected diseases were considered “affected” and those without any disease were considered as “not affected”; individuals with missing data were excluded from the analysis. This variable was included in all statistical analyses to take into account the presence of pathologies that could be correlated to the PGF levels in the study samples.

#### Age, sex, menstruation, and smoking factors

The effect of age, sex, menstruation, smoking, and sex/smoking interaction on the log-transformed PGF was assessed applying a linear regression model with a forward selection. The first predictor tested in the analysis was age. It was considered as a continuous variable. The other terms were then added stepwise in the model, retaining only the significant ones. The Akaike information criterion (AIC) was used to select the best model. All analyses were performed using the R statistical software package version 2.10.0.

To compare the effect of age on PGF levels and the PGF mean values between the two populations, the individuals aged from 40 to 75 years were selected from the Cilento sample to match the range of age covered in the Denmark sample. We refer to this sample as the Cilento “age-matched sample” (N = 464). The estimated coefficients (β) for age in the linear regression performed on the Cilento age-matched and Denmark samples respectively represent the increase of the log-PGF per year. An analysis of variance was carried out to evaluate if the means from the two samples (Cilento age-matched and Denmark) statistically differed using age, sex, menstruation, smoking and *“disease status”* as covariates. Correlations between environmental factors were assessed using an analysis of variance for quantitative variables and a chi-squared test for qualitative variables.

#### Heritability

The heritability of PGF was estimated based on the genealogy data from the Cilento sample using SOLAR software [22]. The age, sex, menstruation, smoking and sex/smoking interaction were tested as covariates and all were retained in the final model. The estimation of heritability was 0.437.

#### Association analysis and Correction for multiple testing

Five SNPs were tested for association with the log-transformed PGF in the Cilento sample. This analysis was performed including each SNP as a predictor factor in the regression model together with the selected environmental factors (age, sex, menstruation, smoking, and sex/smoking interaction) and adjusting for the disease status of the individuals. Only the SNPs with a statistical significance after correction for multiple testing (p-value<0.0127) were selected to be replicated in the Denmark sample and to be included in the best fitting model of this sample.

In the Cilento sample, to account for the inter-individual relatedness of the participants in the association analysis, a linear mixed model was fitted with the lmekin function from the kinship package in R. The lmekin function allows the user to input the kinship matrix as a random effect in the linear mixed model. The genealogical information, drawn from the pedigree, was used to estimate the kinship matrix. In the Denmark sample, including unrelated subjects only, a normal linear model was applied.

To generate the p-value threshold corrected for multiple testing, we applied the procedure of Nyholt [23] modified by Li and Ji [24]. Briefly, a number of independent tests (Meff) equivalent to the number of correlated SNPs tested was estimated from the Linkage Disequilibrium (LD) pattern among the SNPs and a Bonferroni correction for Meff tests was applied. To account for the inter-individual relationships in the Cilento sample, the measurements of LD were computed in a sub-sample of poorly related individuals (N = 250).

A fixed effect meta-analysis using weighted z-scores was used to combine results across the two study samples using the METAL program [25]. For each SNP a reference allele was identified and a z-statistic summarizing the magnitude of the p-value for association under an additive model, along with the direction of the effect, was generated for each study. An overall p-value was calculated using a weighted average of the individual statistics for each sample.

#### PGF risk score

In order to represent the cumulative effects of the environmental and genetic factors, we created a PGF risk score comprising information from the genetic (the associated SNP) and non-genetic factors, following [26]. The risk score was computed for each subject by multiplying the value of each variable by the corresponding β coefficient estimated from the multivariate regression analysis and summing over all the genetic and non-genetic variables. The following equation was applied to calculate the PGF risk score: Rs_i_ = [(β_age_*age_i_)+(β_sex_*sex_i_)+(β_me_*menstruation_i_) (β_smoking_*smoking_i_)+(β_sex:smoking_*sex:smoking_i_)+(β_snp_*snp_i_)] where: i = ith individual, Rs_i_ = Risk score value for individual I, β_age_ coefficient estimated for age, β_sex_ coefficient estimated for sex, β_me_ coefficient estimated for menstruation, β_smoking_ coefficient estimated for smoking, β_sex:smoking_ coefficient estimated for sex/smoking interaction, β_snp_ coefficient estimated for SNP.

To make the cumulative risk score easier to interpret, we rescaled it to range from zero (low PGF levels) to 100 (high PGF levels). Individuals were categorized into 7 classes of risk scores to better describe the PGF variability in the samples.

## Results

The basic features of the population samples as well as the distribution of PGF values within individual cohorts and stratified by sex are reported in Table 1. Respectively, 63% and 52% of the study participants are women in the Cilento and Denmark samples. The mean age in Cilento is higher in women than in men (p = 9.5×10^−9^), a difference not observed in the Denmark sample (p = 0.55). The age range is considerably larger in Cilento (15–103 years) than in the Denmark sample (41–73 years). The proportion of men among smokers is high in both samples but particularly in the Cilento sample: 63% of smokers are men in the Cilento sample (p = 2.1×10^−24^) and 56% in the Denmark sample (p = 2.1×10^−15^. The smokers are older than the never smokers in men. The opposite is true for women (Table 1). This difference is statistically significant in Cilento (p = 9.5×10^−10^) but not in Denmark (p = 0.71).

**Table 1:** The baseline characteristics of the samples.

	Cilento			Denmark		
	All	Men	Women	All	Men	Women
***# participants***						
**All**	871	319	552	1812	873	939
**Menstruation (Pre-menopausal,%)**	-	-	43.5	-	-	39.8
**Smoking**	243	154	89	1110	617	493
**Never Smoking**	628	165	463	702	256	446
***Age, years***						
**All**	49.8 (20.7)	44.6 (18.2)	52.9 (21.4)	54.1 (10.4)	54 (10.3)	54.3 (10.6)
**Smoking**	44.6 (17.2)	47.3 (17.4)	39.9 (15.8)	54.0 (10.4)	54.8 (10.3)	53.1 (10.4)
**Never Smoking**	51.9 (21.5)	42.1 (18.6)	55.3 (21.4)	54.3 (10.6)	52.1 (10.1)	55.6 (10.6)
***PGF, pg/ml*** *						
**All**	12.2 (4.7)	12.4 (4.4)	11.9 (4.9)	15.9 (4.9)	16.5 (4.9)	15.4 (4.8)
**Smoking**	11.8 (4.9)	12.9 (5.3)	10.2 (3.3)	16.3 (5.0)	16.8 (5.1)	15.6 (4.9)
**Never Smoking**	12.3 (4.6)	12.2 (3.8)	12.3 (5.1)	15.4 (4.5)	15.9 (4.1)	15.1 (4.7)

The mean and the standard deviation (SD) are reported for the age, the median and the interquartile range (IQR) for the PGF levels.

* Plasma levels for Cilento and serum levels for Denmark.

### Effect of age, sex, menstruation, and smoking on PGF plasma levels

**Figure 1:**
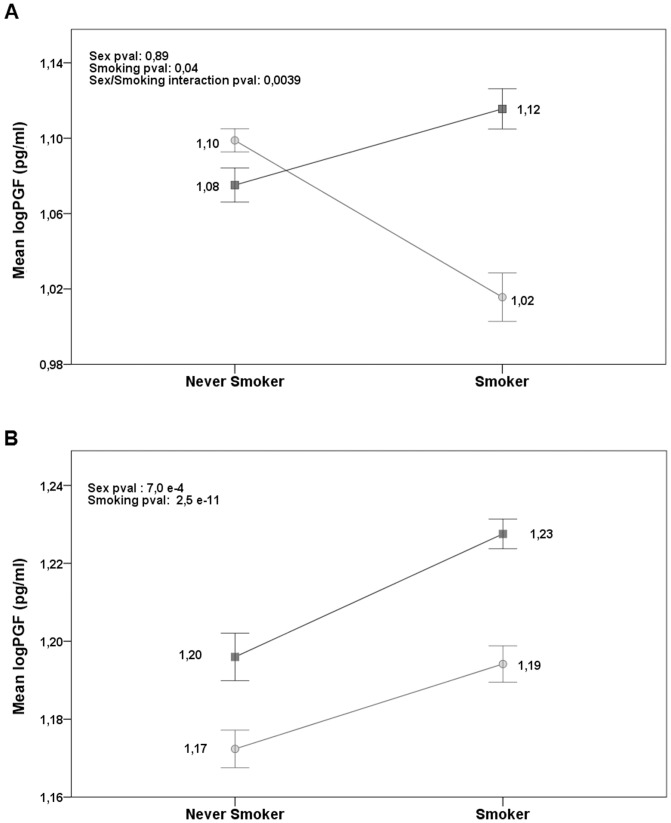
PGF level according to smoking and sex in Cilento and Denmark. Mean levels of the log-transformed PGF and the standard error are reported. A linear regression model includes the log-PGF as a dependent variable, age, sex, menstruation, smoking, sex/smoking interaction, and “disease status” as independent variables for the Cilento sample (A) and age, sex, menstruation, smoking, and “disease status” as regressors for the Denmark sample (B). The p-values for sex, smoking and sex/smoking interaction are shown.

With regard to the environmental factors (age, sex, menstruation, and smoking) analyzed, a forward selection was carried out to select the best-fitting model for the Cilento and Denmark data. As evident in Figure 1, the model including age, sex, menstruation, smoking, and sex/smoking interaction provided a better fit to the Cilento data. In contrast, the best model for the Denmark data involved only the age, sex, menstruation, and smoking factors. The effects of each environmental factor with the relative standard error and the corresponding p-value for the two regression models are shown in Table S1.

In detail, a significant increase in PGF plasma levels with ageing was detected in the Cilento sample (p = 4.2×10^−21^) confirming a result that we recently reported in a selected adult sample [27]. Interestingly, the same strong association between age and PGF plasma levels was also observed in the Denmark sample (p = 4.9×10^−44^).

In consideration of the huge difference in the age range between the Cilento and Denmark samples we selected from the Cilento sample an age-matched subset of individuals covering the same age range as the Denmark sample (the Cilento age-matched sample) to compare the age effect between the two samples. A similar age effect was obtained in the two samples: Cilento age-matched: β (standard error, se) = 0.0037 (0.0005), Denmark: β (se) = 0.0042 (0.0002), where β is the estimated coefficient for age in the univariate linear regression.

**Figure 2:**
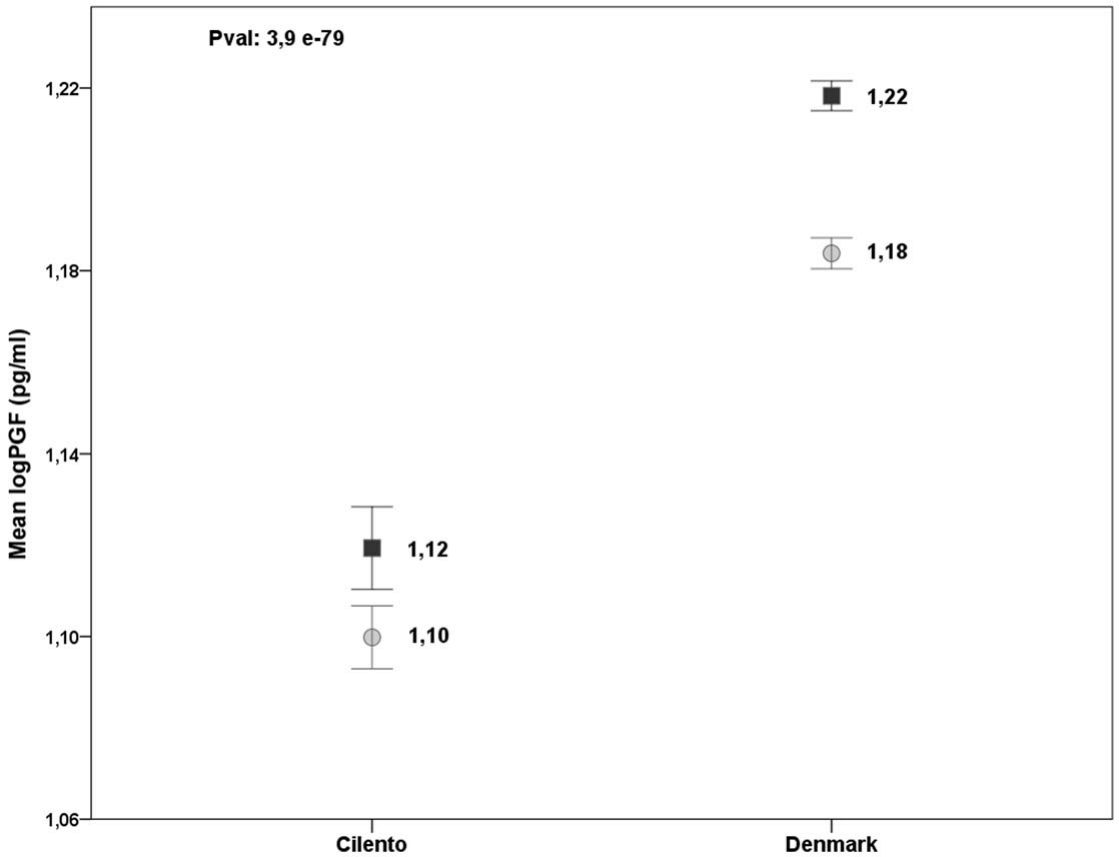
PGF levels according to sex in the Cilento and Denmark samples. Mean levels of the log-transformed PGF and the standard error are reported for each gender and population sample. A univariate analysis of variance including the log-PGF as a dependent variable, sex and village as fixed factors and age, menstruation, smoking and “disease status” as covariates was performed. The corresponding p-value for the PGF level difference between the population samples is shown in the left corner of the plot.

Sex was also found to influence PGF levels. In fact, in Denmark the levels of PGF were more elevated in men than in women (p = 7.0×10^−4^, Table 1, Table S1, Figure 2); in Cilento the effect of sex was observed through its interaction with smoking (Table S1 and Figure 2). Menstruation was also found to have an effect on PGF levels (Table S1). In detail, levels of PGF were lower in pre-menopausal women in comparison to post-menopausal women in which, in turn, the levels were comparable to those observed in men (in Cilento: 10.5±2.8 pg/ml in pre-menopausal women *vs* 14.6±4.2 pg/ml in post-menopausal women *vs* 14.5±4.7 pg/ml in men; in Denmark: 13.8±3 pg/ml vs 17±3.5 pg/ml *vs* 17.2±3.7 pg/ml).

Smoking significantly increased PGF levels in the Denmark sample (p = 2.5×10^−11^) and this increase was evident both in men and in women. An effect of smoking on PGF levels was also detected in the Cilento sample (p = 0.041), but with a different direction according to sex (Table S1). Indeed, stratifying on sex, we observed levels of PGF higher in Cilento smoking men than in never smoking men (p = 3.9×10^−3^) but the levels of PGF were higher in Cilento never smoking women than in smoking women (p = 4.6×10^−8^). This interaction between smoking and sex was significant (p = 3.9×10^−3^) as presented in Figure 1.

As shown in Table 1, the PGF levels are higher in the Denmark sample (median ± interquartile range, sd: 15.9±4.9 in Denmark vs 12.2±4.7 in Cilento) and the difference is highly significant (p = 3.9×10^−79^) (see Figure 2), as assessed with a multivariate analysis taking into account the effect of age, sex, menstruation, and smoking and using the Cilento age-matched sample.

### PGF gene polymorphism association study

The five SNPs located in the *PGF* gene region were: the rs12411, rs8185 and rs2268613 polymorphisms placed in the 3′ regulative region; the rs2268614 variant located in intron 3 and the rs11850328 variant located in the region upstream the *PGF* gene. In the Cilento sample, all the SNPs had a minor allele frequency (MAF) above 5% and were tested for association with the PGF plasma levels. Each SNP was added as a predictor variable in the best-fitting model including the environmental factors (Table 2).

**Table 2:** Association results between the SNPs in the *PGF* gene and the protein levels according to the best fitting models for the Cilento and Denmark samples.

SNPs (*)	HapMap†	Cilento‡			Denmark			Combined§
	MAF	MAF	Effect (SE)	P-value	MAF	Effect (SE)	P-value	P-value
***rs11850328 (T)***	0.38	0.35	0.014 (0.006)	1.8 10^−2^	0.44	0.017 (0.003)	**4.4 10^−9^**	**7.5 10^−10^**
***rs2268614 (A)***	0.38	0.32	0.016 (0.006)	**9.9 10^−3^**	0.44	0.017 (0.003)	**5.2 10^−9^**	**3.6 10^−10^**
***rs12411 (A)***	0.18	0.11	0.006 (0.009)	0.51	-	-	-	-
***rs8185 (G)***	0.17	0.10	0.019 (0.012)	0.087	-	-	-	-
***rs2268613 (C)***	0.18	0.13	0.0002 (0.008)	0.98	-	-	-	-

Association for the five *PGF* SNPs and the protein levels is reported for Cilento; only the SNPs associated in Cilento were tested in Denmark. Statistical associations are all adjusted for age, sex, menstruation, smoking, sex/smoking interaction (only for Cilento) and “disease status”.

* Minor allele referred to the reverse strand according to *PGF* position.

† The CEU sample was chosen as reference population of the HapMap data.

‡ Test corrected for relatedness between individuals. Multiple testing p-value threshold = 0.0127. Significant results are given in bold.

§ P-value from meta-analysis (see Material and Methods section for details).

Significant association was found between the PGF plasma levels and the rs2268614 variant, association close to the significance threshold was detected between the PGF levels and the rs11850328 variant (Table 2). The AA genotype of the rs2268614 variant was associated with higher levels of PGF (mean PGF levels for genotypes AA = 13.0±4.1 pg/ml *vs* GG = 12.5±4.0 pg/ml) whereas the TT genotype of the rs11850328 variant was associated with higher PGF levels (mean PGF levels for genotypes TT = 13.0±4.7 pg/ml *vs* CC = 12.5±4.1 pg/ml). These two associations were then strongly replicated in the independent and larger Denmark sample (Table 2). A meta-analysis of the two samples (N = 2,683) yielded global p-values of 7.5×10^−10^ and 3.6×10^−10^ respectively (Table 2).

The two associated SNPs were common in both populations and, interestingly, the frequencies of the associated alleles were significantly higher in Denmark than in Cilento (for rs11850328, MAF = 0.44 *vs* 0.35, p = 1.7×10^−8^) (Table 2), a result consistent with PGF levels being higher in Denmark (see Figure 1).

A strong LD was observed between the associated SNPs in both population samples (r2 = 0.88 in Cilento and r^2^ = 0.94 in Denmark). Consequently, the additional analyses described below were reported for the rs11850328 SNP only, but comparable results were observed for the rs2268614 SNP.

### PGF risk score

To evaluate the variability of PGF plasma levels according to the cumulative effects of genetic and environmental factors, we created a PGF risk score based on the best fitting model for the Cilento and Denmark samples.

The percentage of the PGF variance explained by these models ranged from 20.3% for the Cilento data to 26.2% for the Denmark data.

**Figure 3:**
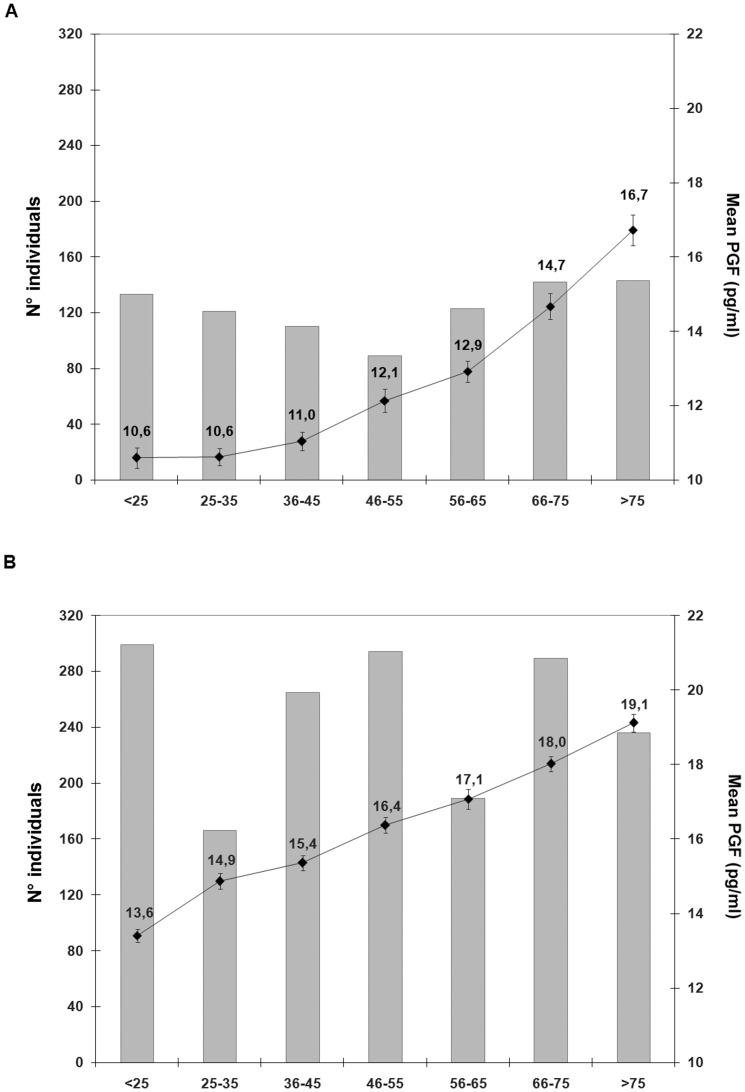
Cumulative effect of the environmental and genetic factors on the PGF levels. Mean PGF levels (right vertical axis) are shown as solid black dots connected by solid lines for categories of the cumulative risk score. The standard error is reported as error bar. The shaded bars show the distribution of the cumulative risk score in the whole population (left vertical axis) in the Cilento (A) and Denmark (B) sample.

In Figure 3, the average PGF levels across the different risk score classes are shown both for the Cilento (A) and Denmark sample (B). For the Cilento sample, the individuals in the highest score group (HSG) had the mean PGF levels (16.7 pg/L; sd: 4.9) that were 1.6 times the levels observed for individuals in the lowest score group (LSG) (10.6 pg/L; sd: 3.2), while, in the Denmark sample, we observed an increase of the mean PGF levels of 1.4 times between the two extreme score groups (HSG: 19.1 pg/ml, sd:3.7 *vs* LSG: 13.4 pg/ml, sd:4.0).

## Discussion

In this work, we have demonstrated, for the first time, that PGF plasma levels are influenced by both genetic and environmental factors. We conducted the study in two independent population samples: a homogeneous sample of isolated populations from Cilento, South Italy, and a European general population sample from Denmark.

Focusing on non-genetic factors, the principal predictor element is age that alone explains 16% of the variability of PGF in the Cilento and 19% in the Denmark sample. Furthermore, the log-transformed PGF levels were estimated to increase by about 0.4% per year (Cilento: 0.37%, Denmark: 0.42%, matched for age range).

Regarding the significant sex effect observed, it could be mediated by hormones. Indeed, variations in PGF concentration with the phases of the menstrual cycle have been described [28] and the regulatory effects of the estrogens on PGF production have been observed in pregnancy [29]. Moreover, in our study an increase of PGF levels was observed in the post-menopausal women in comparison to pre-menopausal women. Interestingly, in post-menopausal women PGF reached levels comparable to those observed in the male sample both in Cilento and Denmark. However, the interaction between sex and smoking observed in Cilento cannot be explained by menstruation.

The effect of smoking is somewhat more complex. Indeed, while an increasing effect on PGF levels was observed in all men and in Danish women, an opposite decreasing effect was observed in Cilento women (Figure 1). The smoking intensity between women in Cilento and Denmark was explored. However, we were able to exclude the smoking burden as a possible explanation of the PGF level difference between the two female samples (data not shown).

In literature, divergent findings have been reported regarding the effect of tobacco smoke on angiogenesis. Depending on the smoke compound analysed, opposite effects have been observed. In fact, some studies have demonstrated that different tobacco smoke compounds such as 2,3,7,8-tetrachlorodibenzo-p-dioxin (TCDD), pyridine and pyrazine derivatives may lead to an inhibition of angiogenesis [30], [31]. On the other hand, it has been firmly demonstrated that nicotine has a pro-angiogenic effect. Several angiogenic factors have been found to be enhanced by nicotine. The expression of three relevant pro-angiogenic molecules (basic fibroblast growth factor, bFGF, platelet-derived growth factor, PDGF and vascular endothelial growth factor, VEGF), is induced by nicotine in cancer [32]. Conklin et al. [33] also described how both nicotine and cotinine, at doses similar to those seen in habitual smokers, significantly increased the endothelial cell expression of VEGF, stimulating angiogenesis with worse consequences on atherosclerosis and tumour growth. In light of these data, the hypothesis that an environmental factor specific to the Cilento women and yet to be identified could inverse the effect of cigarette smoke on PGF levels does not seem irrelevant. But taken together, these data suggest that cigarette smoking may have an effect on angiogenesis by modulating the expression of PGF and that this effect may be buffered by other factors, yet to be identified.

We have observed diverse PGF levels in the two population samples. Similar results have been described for other circulating proteins such as VEGF [34]. Also, Oggè G. et al reported comparable levels of PGF in both the plasma and serum of women with a normal pregnancy in the first, second and third trimesters [35] suggesting that the detected difference in our population data may not be ascribable to the source of the PGF measurements. Moreover, in our work, we have found that the environmental and genetic factors have a similar effect in both study samples suggesting that the difference in median levels between the two populations could be related to the effect of other factors able to influence the PGF levels. Nevertheless, different storage conditions cannot be excluded as a possible cause of the difference in the PGF levels.

Furthermore, taking advantage of the genealogy available for the Cilento sample, we observed that the PGF plasma variation is a heritable trait with a heritability of 0.437, a value comparable to many quantitative traits analyzed in genetic studies [36]. Through an association analysis carried out in the *PGF* gene region, we found that in Cilento two SNPs (rs2268614 and rs11850328) were associated with the levels of the protein in the plasma and this result was strongly replicated in the independent population sample from Denmark. The rs2268614 polymorphism is located in intron 3 and the rs11850328 is placed upstream the gene, 4.3 kb from the transcription starting site. Given that the LD between the two SNPs is very high, two alternative hypotheses could be formulated about the association results: 1) one of the two identified SNPs has a functional role on the variation of PGF; 2) both SNPs are in LD with another unidentified causal variant. Upholding the first hypothesis, a bioinformatics approach was used to gain information about any evidence of a possible functional role of these variants.

Neither of the two SNPs was reported as eSNP for the *PGF* gene. However, exploring the eQTL database (Genevar, http://www.sanger.ac.uk/resources/software/genevar/), both variants were found to be eSNPs for the *EIF2B2* and *MLH3* genes [37], [38], [39]. Both genes were located in the same cytogenetic band of *PGF* (14q24.3), close to the *PGF* gene region the *MLH3* gene was on the same strand of *PGF*, *EIF2B2* on the other strand. No evident functional links between EIF2B2 or MLH3 and PGF have been found so far.

An analysis of potential transcription factor binding sites by using the Mat-Inspector Online Tool (http://www.genomatix.de/) was also performed. This analysis showed that the rs11850328 polymorphism is located in the core enhancer sequence for a TEA/ATTS DNA binding domain factor. This protein belongs to a highly conserved protein family whose expression patterns have been correlated with the transcription of viral genes [40] and which is considered essential for cardiac, skeletal and smooth muscle development [41]. In particular, a member of this family, TEF-5, is expressed in the placenta and influences foetal development [42]. The same analysis carried out for the rs2268614 SNP also revealed a potential binding site at the SNP position: in fact, the variant is placed in the core sequence of the binding site for the GA-binding factors. Therefore, both SNPs seem to be good candidates as causal variants modulating PGF levels; nevertheless functional studies are required to demonstrate if a direct involvement of these SNPs in the PGF variation exists.

Looking at the cumulative effect of genetic and environmental factors on the population samples we were able to explain about 23% of the PGF variation. In addition, by introducing PGF risk score groups, a more detailed characterization of each population sample could be defined. In fact, the risk group distributions of the two samples appeared globally different. Moreover, the Denmark sample appeared to be not equally distributed among the different risk groups although no difference in the number of individuals was observed between the low-risk and high risk classes. Also, the distribution of PGF mean levels gradually increases across the risk groups. In the Cilento sample, individuals seem to be more homogenously distributed among the different risk groups although, in this case, the high-risk classes are slightly more abundant compared to the low-risk classes. Furthermore, as a consequence of the best-fitting model for this population sample, no uniform increase of the PGF mean levels across the risk groups can be observed. In particular, a consistent steep rise of the PGF levels is evident in the last two classes in comparison to the rest of the distribution.

Several studies have described PGF plasma levels as a potentially powerful clinical biomarker of vascular inflammation and an adverse outcome in patients with acute coronary syndromes [43], [44] and as an independent predictor of coronary heart diseases [16]. We have also recently shown a significant increase of PGF in the Metabolic syndrome [27]. However, the present study reveals that the effect of genetic and environmental factors on the circulating levels of PGF is independent of possible PGF-correlated pathologies such as cardiovascular and inflammatory diseases.

In conclusion, this work illustrates that genetic variants, age, sex, menstruation, and smoking, influence PGF variability in the Cilento and Denmark samples and reveals a significant sex/smoking interaction in the Cilento sample. Further studies are required to investigate the influence of additional factors able to influence the PGF levels in these populations, in particular in the Cilento sample.

## Supporting Information

Table S1 **Association results between non genetic factors and the PGF levels according to the best fitting models of the Cilento and Denmark samples.**
(DOCX)
