# Supplementary material for: Genetic and Environmental Factors Influencing the Placental Growth Factor (PGF) Variation in Two Populations
Source: PLoS One. 2012 Aug 20;7(8):e42537. doi: 10.1371/journal.pone.0042537 (PMC3423400; doi:10.1371/journal.pone.0042537)
Supplement: Table S1 — Association results between non genetic factors and the PGF levels according to the best fitting models of the Cilento and Denmark samples. (DOCX) [file pone.0042537.s001.docx]

**Supplementary table 1.** Association results between non genetic factors and the PGF levels according to the best fitting models of the Cilento and Denmark samples.

| **Variables** | **Cilento^#a^** | | | **Denmark^b^** | | |
| --- | --- | --- | --- | --- | --- | --- |
|  | **Effect** | **SE*** | **P-value** | **Effect** | **SE*** | **P-value** |
| **Age** | 0.0030 | 0.0003 | **4.2 10^-21^** | 0.0035 | 0.0002 | **4.9 10^-44^** |
| **Disease status (Disease)** | -0.0095 | 0.0107 | 0.3764 | 0.0143 | 0.0045 | **0.0015** |
| **Menstruation**  **(Pre-menopausal)** | -0.0336 | 0.0133 | **0.0118** | -0.0317 | 0.0071 | **7.3 10^-6^** |
| **Sex (Male)** | -0.0016 | 0.0125 | 0.8985 | 0.0174 | 0.0051 | **7.0 10^-4^** |
| **Smoking Habit (Smokers)** | -0.0261 | 0.0128 | **0.0411** | 0.0293 | 0.0044 | **2.5 10^-11^** |
| **Sex/Smoking Interaction** | 0.0509 | 0.0176 | **0.0039** | - | - | - |

^*^Standard error

^#^ Test corrected for relatedness between individuals.

Best fitting model: a) Y ~ A + S_x_ + S_h_ + I+ M_e_ + D_s;_ b) Y ~ A + S_x_ + S_h_ + M_e_ + D_s_

(Y = logPGF, A = Age, S_x_ = Sex, S_h_ = Smoking, I = Sex/Smoking interaction, M_e_ =

Menstruation, D_s_ = Disease status)

Significant p-values are given in bold.
